# Molecular Markers in Embryo Non-Development: Analysis of Gene Expressions (*Ki-67*, *hTERT*, *HIF-1α*) in Spent Embryo Culture Medium

**DOI:** 10.3390/cells13242093

**Published:** 2024-12-18

**Authors:** Nergis Özlem Kılıç, Duygu Kütük, Çağrı Öner, Senem Aslan Öztürk, Belgin Selam, Ertuğrul Çolak

**Affiliations:** 1 Department of Histology and Embryology, Medical Faculty, Maltepe University, 34844 İstanbul, Turkey; nergisozlemaltin@gmail.com (N.Ö.K.); duygukutukk@gmail.com (D.K.); senemaslanozturk@gmail.com (S.A.Ö.); 2 IVF Laboratory, Bahçeci Umut Assisted Reproduction Center, 34662 İstanbul, Turkey; 3 Department of Medical Biology, Medical Faculty, Kırklareli University, 39100 Kırklareli, Turkey; cagrioner@klu.edu.tr; 4 Department of Medical Laboratory Techniques, Vocational School, İstanbul Atlas University, 34408 İstanbul, Turkey; 5 Department of Obstetrics and Gynecology, School of Medicine, Acibadem Mehmet Ali Aydinlar University, 34752 Istanbul, Turkey; 6 Department of Biostatistics, Medical Faculty, Eskişehir Osmangazi University, 26040 Eskişehir, Turkey; ecolak@ogu.edu.tr

**Keywords:** differentiation, normoresponder, non-invasive, proliferation, telomerase, spent embryo culture medium

## Abstract

An embryo culture medium is a specialized set of ambient conditions, technological equipment, and nutrients that embryos require to grow properly. We aimed to investigate the *Ki-67*, *hTERT*, and *HIF-1α* gene expression differences between developing and non-developing embryos in spent embryo culture medium. *Ki-67*, *hTERT*, and *HIF-1α* gene expressions were determined from the spent embryo culture medium containing developing and non-developing embryos of 20 normoresponder patients admitted to the Bahçeci Umut IVF Center. An increase in *hTERT* gene expression (*p* < 0.05) and a decrease in *HIF-1α* gene expression (*p* < 0.001) were observed in mediums of developing compared to the non-developing embryos. No difference was observed in *Ki-67* gene expression (*p* > 0.05). While there was a correlation between *Ki-67* and *HIF-1α* genes in the non-growing group (r < 0.01); no correlation was observed in the developing group (r > 0.05). Both normoresponder groups will be similar in terms of proliferation rate. The low *HIF-1α* expression that observed high telomerase activity in embryo development maintains continuity and avoids mechanisms that result in cell death. A molecular study of the embryo development in patients with similar characteristics may help to understand the pathogenesis of the disease and establish a diagnosis and specific treatment.

## 1. Introduction

Mitochondria are the power plant of the cell, and their dysfunction leads to the development of neurodegenerative diseases, cardiovascular pathologies, and various types of cancer [1].

A significant number of genes implicated in pathophysiological processes have their expression regulated by hypoxia, an unavoidable cellular stress in many illnesses [2].

The capacity of tumor cells to adjust to variations in oxygen levels is crucial for tumor progression.

Reduced oxygen levels impact cellular metabolism, hence affecting proliferation, migration, and invasion.

The low oxygen concentration causes an increase in reactive oxygen species in cellular compartments thus effects the down regulation of the cell cycle and survival of the cell. This cellular regulation against to oxygen absence causes cell death and decreases the proliferation. Furthermore, these inappropriate conditions lead to migrating cells to places with more oxygen levels [3]. The focal point of cellular adaptation to hypoxia is the transcription factor hypoxia-inducible factor 1 alpha (*HIF-1α*), which influences the expression of specific gene networks involved in cellular energy and metabolism [4]. Hypoxia activates *HIF-1 α*, a nuclear protein that appears as heterodimers and is involved in transcription. Numerous physiological functions, including the growth of cartilage, the immune system, and the cardiovascular system, depend on it. Additionally, during hypoxia, it can trigger compensatory reactions in cells via upstream and downstream signaling networks. Furthermore, promoting mitochondrial dynamics and *HIF-1 α* activation requires a change in oxygen levels.

While high mitochondrial activity causes high proliferation rate in cancer development, the abnormal mitochondrial membrane potential may cause uncontrolled cell death as neurodegenerative disorders. *HIF-1α* may be a promising target to modulate mitochondrial dynamics to develop therapeutic approaches for NDs, immunological disorders, and other related diseases [5].

The decision of whether a cell will live, divide, differentiate, or die depends on the interactions of intracellular and extracellular factors. Examples for intracellular interactions are genetic and epigenetic mechanisms, while lifestyle radiation and UV rays are the good examples of extracellular interactions. Signals from the external environment are transmitted into the cell by various receptors on the cell surface. The response to the transmitted message is determined by a complex set of factors in the cell’s internal environment [6]. Only specific cell groups inside the body are actively dividing at any given time due to the strict regulation of cell division in mammals. Some cells, however, are able to evade these restrictions, dividing quickly and developing into tumors. *Ki-67* is produced only in actively dividing cells where it is in the nucleus, the structure containing most of the cell’s DNA [7]. *Ki-67* is used as a marker to determine which cells are actively dividing in tissue samples from cancer patients and have shown that Ki-67 is required for cells to divide [8]. Predicted molecular weights of 320 kDa and 359 kDa are associated with two human isoforms of the nuclear DNA-binding protein *Ki-67* [9].

*Ki-67* protein levels and localization change throughout the cell cycle. *Ki-67*, a nuclear protein associated with mitotic activity, is localized in all phases of the cell cycle except the G0 phase [10]. In the G2 phase or during mitosis, the highest expression occurs [11]. The presence of several tandem repetitions (14 in mice and 16 in humans) with a conserved motif of uncertain function, called the “*Ki-67* domain”, is the most distinctive property of *Ki-67*.Two other conserved motifs, a protein phosphatase 1 (PP1) binding motif and a conserved acid motif containing 22 amino acids, 100% identical between human and mouse, conserved in all homologues of unknown function, contain a conserved domain (CD) of 31 amino acids [12]. Cell proliferative capacity in meningiomas has been demonstrated to be effectively determined by *Ki-67* homologues, which have a poorly conserved leucine/arginine-rich C-terminus that can bind to DNA and, when overexpressed, enhance chromatin packing [13]. It is routinely used in clinical practice in meningiomas due to its correlation with the mitotic index and histopathological grade. The *Ki-67* proliferation index (PI) has been reported to have a strong correlation with tumor growth, recurrence, and disease-free survival in various tumors including meningiomas [14].

It has been reported that telomeres are among the mechanisms underlying cell proliferation and consist of catalytically active reverse transcriptase (TERT) and its RNA template TERC, and are G-rich repetitive sequences near the ends of chromosomes that are duplicated by the specialist reverse transcriptase telomerase [15,16]. Human normal somatic cells lack telomerase and are unable to preserve telomeres during cell division because *hTERT* is kept in a closed state. Telomeres thus progressively become shorter until they are so short that they cause apoptosis or replicative senescence [6,17,18]. *TERT* has been reported to be a dominant determinant of telomere length, a relevant biomarker for cellular ageing [19]. *TERT* plays critical functions in various tissues and its dysfunction has been associated with impaired tissue repair or regeneration in various pathological conditions [20]. It has been reported that the highly conserved telomeric DNA sequence (TTAGGG) is maintained through telomerase activation and telomerase dysfunction leads to morphological, biochemical, and functional changes in tissues [19,21,22]. Transgenic expression of *TERT* has been reported to increase the reprogramming efficiency and promote proliferation of primary cell culture and induced pluripotent stem cells. In this study, the cre-loxP system was used to conditionally induce the expression of the *TERT* gene in specific cells; therefore, in this way, *TERT* was observed to prolong the culture time in keratinocytes (skin cells) and increase the colony formation of induced pluripotent cells (iPS). These mentioned findings suggest that transgenic *TERT* expression is effective in the process of transformation into iPS [23]. Female infertility has been reported to result from shortened telomere length in various reproductive tissues [20]. Downstream of *TERT*, mRNA and telomerase activity in GCs surrounding the oocyte have been reported to be one of the main mechanisms involved in female gamete degradation. Telomerase activation or telomerase-based therapies have been developed to improve health span and reduce age-related diseases [21,22].

With the objective to investigate proliferation mechanisms and telomerase activity, this study compared the levels of gene expression for the Ki-67, *hTERT*, and *HIF-1α* genes in spent culture medium samples of developing and non-developing embryos of patients applying to the IVF center. It also aimed to profile the relevant patient groups and make the spent embryo culture mediums which are used in IVF centers and then eventually become useful for molecular diagnosis. According to the hypothesis of this study, the low *hTERT* gene expression and high *HIF-1α* gene expression profiles caused to observe non developing embryos. On the other hand, approximately the same *Ki-67* gene expression indicates that there are not any differences between the groups.

## 2. Materials and Methods

Women who were between the ages of 22 and 43 applied to Bahçeci Health Group Umut IVF Center were included in the study (Ethics Committee No: 2022/20-05). A total of 40 samples were collected from each of the two groups as developing (*n* = 20) and non-developing (*n* = 20) embryos. The collected 40 spent embryo culture medium samples from the normoresponder group were classified into two groups as of developing and non-developing embryos. Anti-Müllerian hormone (AMH) values of 1.0–3.5 ng/mL and an antral follicle count (AFC) of 7–12 were prerequisites for inclusion and the exclusion criteria were AFC count outside the range of 7–12 and AMH value outside the range of 1.0–3.5 ng/mL.

### 2.1. Investigation of Characteristics of SpentEmbryo Culture Medium Samples

**Figure 1:**
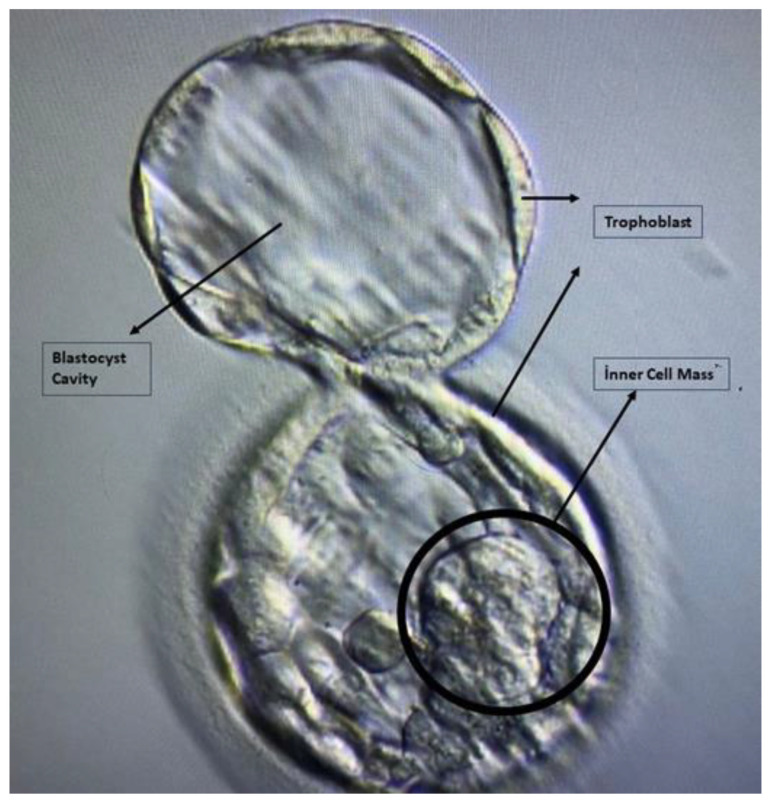
Early development of an embryo at the blastocyts stage.

Gardner’s classification was used to score embryos from culture effluent samples. On days 2 and 3 of embryo development, a cleavage stage morphological score was determined based on a three-stage grading system using characteristics such as cell number, fragmentation, symmetry and morphology. At the blastocyst stage (day 5–6), the morphological score was determined based on the expansion stage, the quality of the inner cell mass (ICM), and the quality of the trophectoderm [24] (Figure 1). The criteria for the Gardner’s classification is shown in Table 1.

### 2.2. Collection of Culture Medium Samples Containing Human Embryos

**Figure 2:**
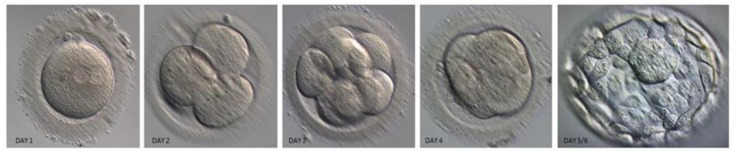
According to the Gardner’s classification, each blastocyst which was monitored on day 3 and 5–6 after fertilization, their embryo expansion, internal cell mass (ICM), and density of trophoectoderm (TE) cells were evaluated and then spent culture fluid was collected.

**Figure 3:**
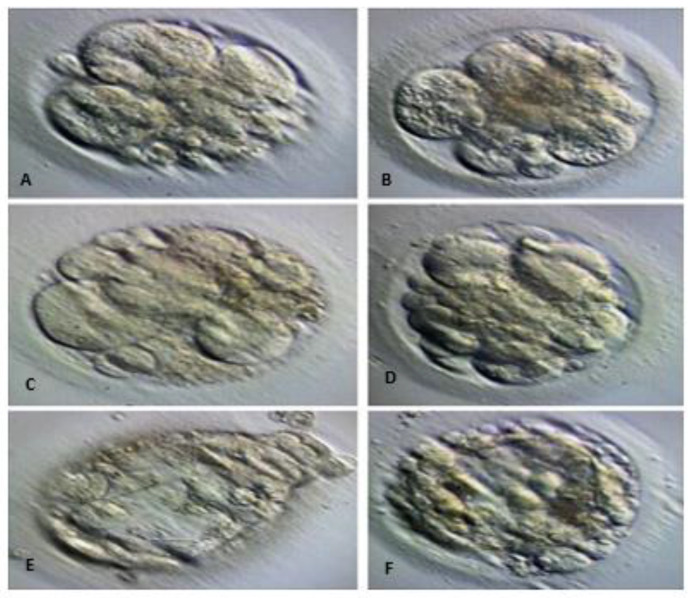
Until day 6, spent culture fluid was collected from non-degenerating embryos that were followed up at the blastomere level or at the arrest level in the first stage of blastocyst development, where degeneration started. (**A**–**D**): day 3 embryo whose blastomeres continue to be counted, fragmentation is observed and regeneration begins. (**E**,**F**): arrested day 5 embryo in which the blastocoel cavity is beginning to form, the trophoectoderm structure and (ICM) structure are not clearly visible and degeneration has begun.

The culture medium containing the embryos frozen on days 5 and 6, which were checked for development in the laboratory and then discarded, was analyzed (Figure 2 and Figure 3). The spent culture fluid samples from the embryo culture drops were collected into PCR tubes using The Stripper™ Pipettor and Tips (CooperSurgical, Trumbull, CT, USA) were stored at −80 °C for later analysis [25].

### 2.3. Total RNA Isolation from Human Spent Embryo Culture Medium and Determination of Gene Expression by Real Time Polymerase Chain Reaction

Following the manufacturer’s instructions, total RNA was extracted using a Nucleospin RNA Kit (InnuPREP MicroRNA Kit, Jena, Germany), and cDNA was created by reverse transcription of total RNAs (Nucleogene, Istanbul, Turkey). The cDNA reverse transcription was performed at 42 °C for 60 min and 95 °C for 5 min. SYBR Green primer sets for the amplification of *Ki-67*, *Hypoxia Inducible Factor-1 Alpha* (*HIF-1α*), *Human Telomerase Reverse Transcriptase* (*hTERT*), and *Glyseraldehide-3-phosphate ehydrogenase* (*GAPDH*) were designed and supplied by Bmlabosis (Ankara, Türkiye). The primer sequences are shown in Table 2. *Glyceraldehyde-3-phosphate dehydrogenase (GAPDH)* was used as an internal control for the calculation of ΔCT values [26] (Table 1).

### 2.4. Statistical Evaluations

All statistical evaluations were performed using IBM SPSS Statistics 26.0 package program. Normal distribution of continuous variables was obtained using the Kolmogorov-Smirnov compatibility test. Comparisons between normally distributed variable groups were evaluated using one-way analysis of variance. Comparisons between non-normally distributed variable groups were evaluated using the Kruskal–Wallis test. Multiple comparisons of gene expressions were compared using Student’s *t*-test. Correlations were determined by the Pearson correlation test. The data obtained were written as mean ± standard deviation (sd) in the text. Significance levels between groups (*p* < 0.05; *p* < 0.01; and *p* < 0.001 or *p* > 0.05) were determined.

## 3. Results

**Figure 4:**
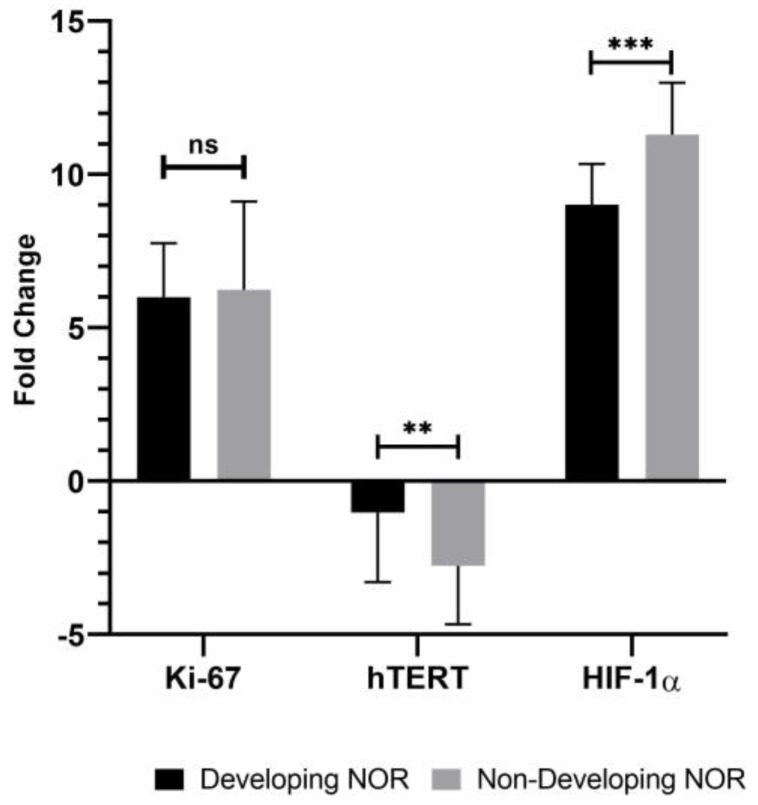
The fold change of *Ki-67* (*p* > 0.05), *hTERT* (*p* < 0.05), and *HIF-1α* (*p* < 0.001) gene expressions which were determined in developing and non-developing embryo spent culture medium from normoresponder (NOR) patients (ns indicates *p* > 0.05 as non-significance; ** indicates *p* < 0.05; and *** indicates *p* < 0.001).

According to the data obtained, no statistical difference was observed in *Ki-67* gene expression in the spent embryo culture medium of developing embryos of normoresponder patients (5.998 ± 1.758) compared to the samples in the spent embryo culture medium of non-developing embryos (6.235 ± 2.879) (*p* > 0.05; Figure 4). A statistically significant increase was observed in *hTERT* gene expression in the spent embryo culture medium of developing embryos of normoresponder patients (−1.022 ± 2.274) compared to the samples in the spent embryo culture medium of non-developing embryos (−2.759 ± 1.904) (*p* < 0.05; Figure 4). A statistically significant increase was observed in *HIF-1α* gene expression in the spent embryo culture medium of developing embryos of normoresponder patients (9.01 ± 1.335) compared to the samples in the spent embryo culture medium of non-developing embryos (11.298 ± 1.688) (*p* < 0.001; Figure 4, Supplementary Materials S1).

No correlation was observed between the genes analyzed in the spent embryo culture medium of developing normoresponder embryos (r > 0.05; Table 3). A low correlation was observed between *Ki-67* and *HIF-1α* genes (r < 0.01) in spent embryo culture medium containing non-developing normoresponder embryos. However, no correlation was observed between *hTERT* and other genes (r > 0.05; Table 3).

## 4. Discussion

Reduced fertility, increased aneuploidy in oocytes and early embryos, and poor developmental outcomes that may be linked to long-term health hazards for offspring are all known effects of advanced maternal age (AMA). However, natural aging complicates research into the underlying effects of AMA on embryo developmental capacity, which further impacts the success of reproduction. In a study evaluating the effect of AMA on mouse blastocyst development, a mouse embryonic stem cell (mESC) model was established to investigate the effects of AMA on the incidence of aneuploidy and developmental potential. Young (7–8 weeks old) and aged (7–8 months old) C57BL/six female mice were mated with young males. Developmental delay in blastocyst morphogenesis was observed in these preimplantation embryos. Advanced maternal age was reported to further reduce mESC survival and proliferation and decrease expression of the cell proliferation marker *Ki-67* [27]. In a study looking at follicle diameter and survival rate, estrogen production, and expression of folliculogenesis-related genes, follicles isolated from 10 to 12 day old mice were cultured and immunohistochemical staining for CD34, *Ki-67*, and CD45 was performed to characterize these cells. At the end of the study, it was reported that atrophy of the reproductive organs was effectively ameliorated and at the same time prevented the increase in body weight and rectal temperature [28]. Normoresponder patients are patients who have a normal oocyte count but do not develop fertility for an unknown physiological, biochemical, and/or genetic reason. Since the characteristics of the patient group did not change in individuals with a normal number (seven or more) of oocytes and their embryos, it was not considered that there would be a difference in proliferation rates of developing and non-developing samples. The low-*hTERT* and high-*HIF-1α* gene expression may be the cause of the morphological abnormalities observed in the non-developing embryo of the patient’s samples that were collected. Ki-67 is a gene region indicating cell proliferation; the nonsignificance of this gene abundance between the groups is an expected result because normally the patient syndrome that makes up the groups is the same syndrome, hence *n* ≥ 7 embryos. *Ki-67* gene expression data focus on this parameter. Considering the data obtained, no difference was observed between the groups in *Ki-67* gene expression in RNAs isolated from the embryo spent culture fluid of developing and non-developing patients.

Female fertility is characterized mainly by the pool of ovarian follicles, both in terms of quantity and quality. The mitotic index of human cells is significantly influenced by telomere length. Consequently, women’s fertility may be directly impacted by telomere homeostasis disruption. The best way to prevent telomeric attrition and preserve ovarian reserve is to use telomerase, which is highly expressed in the ovaries. We have been investigating this issue, hoping to find long telomeres and strong telomerase activity in PCOS and short telomeres and low telomerase activity in POF patients. Although the results of these studies are conflicting, the effect of telomere length and ovarian telomerase on fertility disorders in women seems to be clear [28]. Telomere length and telomerase activity in ovarian cells play significant roles in female fertility. Telomerase is an enzyme that helps maintain telomere length, which is essential for cellular longevity and reproductive health. During folliculogenesis, telomerase activity varies across different stages of follicle development and is generally higher in smaller, healthy follicles and in the oocytes within preantral and preovulatory follicles. This high telomerase activity supports the maintenance of oocytes, making them viable for potential fertilization [29]. Aging has been reported to significantly affect ovarian telomerase activity in bovine, leading to a decrease in the proliferative activity of granulosa cells [30]. Ovarian *TERT* expression in mice is found to decrease with aging [31]. It has also been shown that ovarian *TERT* declines with age in humans and could be linked to telomere degradation. [32]. Women with PCOS who were undergoing intracytoplasmic sperm injection (ICSI) for in vitro fertilization had their ovarian cells’ telomere length and telomerase activity assessed by Pedroso et al. in comparison to women with regular monthly cycles. They found no difference between women with and without PCOS in terms of cumulus cell telomere length. Even though the PCOS group had increased telomerase activity in cumulus cells and immature oocytes (germinative vesicles and metaphase I), the two groups did not differ in telomere length or telomerase activity [33]. According to Kinugawa et al., telomerase is active in the ovaries and often declines with age and in PCOS patients who have follicular depletion [34]. Compared to healthy controls, women with PCOS have been found to have shorter telomeres and reduced telomerase activity in granulosa cells. These studies suggest that short telomeres may be associated with reduced fertility and shorter reproductive lifespan [29,35].

*hTERT*, also known as telomerase enzyme, is the main enzyme that causes high proliferation, i.e., high rates of cell division, especially in cancer cells. Decreased activity of this enzyme is also seen in aging and aging-related degeneration. In other words, a decrease in *hTERT* gene expression is expected in cases where degeneration and related cell deaths increase and cellular proliferation decreases. The *hTERT* is the catalytic subunit of telomerase, which is essential for maintaining telomere length and cellular proliferation. Geifman-Holtzman et al. reported that hypoxia can enhance *hTERT* expression, potentially as a compensatory mechanism to counteract telomeric damage during early pregnancy. *hTERT* may play a protective role in reproductive tissues under stress conditions, including hypoxia. The interplay between *HIF-1α* and *hTERT* is also evident in the context of oxidative stress, which is a common consequence of hypoxia. Li et al. discussed how excessive reactive oxygen species (ROS) can lead to germ cell apoptosis and impaired fertility, with *HIF-1α* mediating these effects [36].

In in vitro fertilization, it is expected to be higher in developing embryos on days 5 and 6, which are the days when cell number increases due to embryonal development. In non-developing embryos, it is theoretically thought that the cell number may decrease instead of increasing. In the light of the data obtained, it was determined that telomerase enzyme activity was higher in the waste culture fluids of developing embryos than in non-developing embryos. This suggests that low expression of the *hTERT* gene may be the reason for the lack of development of non-developing embryos at the molecular level in normaresponder cases. Conversely, high *hTERT* gene expression was also observed in developing embryos.

A benign gynecological condition, endometriosis, affects roughly 10% of women who are of reproductive age. The quality of life is significantly diminished in endometriosis patients due to their chronic dysmenorrhea, dyspareunia, and possibly infertility. Blocking the hypoxic effect is thought to be the best therapeutic approach for the treatment of endometriosis because it is a potent regulator in the disease’s etiology. Targeting the *HIF-1α* protein in endometriosis mice has been shown in several studies to effectively decrease vascular permeability and hence suppress lesion growth [37]. Numerous genes implicated in the pathogenic circumstances of endometriosis were shown to be directly regulated at the transcriptional level as a result of the action of *HIF-1α* stabilized during hypoxia [38]. Therefore, it has been reported that targeting the hypoxia-mediated gene regulatory network as a therapy in clinical treatment may be a promising strategy in the future [39]. In a study investigating the role of mitophagy regulated by *HIF-1α* under hypoxic conditions, it was reported that silencing of *HIF-1α* suppressed the expression of mitophagy-related genes and resulted in higher mortality under hypoxic conditions [40]. In a systematic study of oxygen responses in preimplantation embryo development, it was observed that *HIF-1α*, as a maternally accumulated oocyte factor, may play an important role in genome activation of the major zygote at the cleavage stage after fertilization [41].

*HIF-1α* has recently been recognized as a molecule that helps cells survive in a hypoxic microenvironment. *HIF-1 α* is a crucial transcription factor that controls how cells react to low oxygen tension and hypoxia. During the first and second trimesters, *HIF-1α* primarily contributes to the placenta’s development and differentiation. During normal pregnancy, *HIF-1α* responds to changes in oxygen tension and the release of cytokines and angiogenic factors. Pregnancy-related vascularization and placental function are thought to be significantly influenced by *HIF-1α* [42]. However, recent studies have shown that it is one of the initiator molecules of mitophagy, a mechanism that causes cellular death, namely mitochondrial autophagy [43,44]. Following mitophagy, cells undergo apoptosis. If many mitochondria are mitophagically destroyed from the cell, the cell enters apoptosis and dies. Mitophagy does not result in apoptosis if the number of mitochondria is tolerable, and the mitochondria are able to return to self-synchronization. It is expected that mitophagy and associated apoptosis will not occur in developing embryos. Because if apoptosis is observed, cell number and telomerase enzyme will decrease, and the embryo will not be able to develop [45]. The obtained data also support this hypothesis. Both *HIF-1α* and *hTERT* are involved in several mechanisms that may affect fertility, particularly in the context of hypoxia and oxidative stress. *HIF-1α* is a critical regulator of cellular responses to low oxygen levels and its activation has been linked to various reproductive pathologies. Zhu et al. noted that hypoxia, which is often present in conditions such as varicocele, can induce HIF-1α, which then activates autophagy-related genes such as BNIP3. This process is crucial for maintaining cellular homeostasis in the seminiferous epithelium, and dysregulation can lead to infertility [46]. The high expression of *HIF-1α* gene in the waste culture fluid of non-developing embryos suggests that mitophagy-induced cell death is high, whereas the opposite was observed in the waste fluid of developing embryos, suggesting that mitophagy is suppressed. Considering the findings, overexpression of the HIF-1α gene may be one of the molecular reasons for the failure of embryos to develop in normoresponder cases.

When all the results are evaluated together, we suggest that the high expression profile of *hTERT* and low *HIF-1α* genes may be molecular markers of embryo development in NOR cases. The fact that the number of embryos of patients with the same syndrome is almost the same is an expected result, but the difference in the number of embryos may actually reveal an unexpected result. The lack of significance in this difference is thought to be supportive of the statistical difference observed in *hTERT* and *HIF-1α*. Therefore, no difference between the groups in terms of proliferation and cell number provides certainty in the evaluation of *hTERT* and *HIF-1α* results. The low telomerase activity is an indication of cell senescence, and low *hTERT* expression is a sign of low telomerase activity. However, the increase in *HIF1- α* expression suggests that degeneration is accelerated, even though it is known that degeneration happens under hypoxic conditions. However, a statistical correlation of this situation has not been made, and such a comparison is not among the main objectives of the study. This situation will be taken into consideration in future studies. This study is one of the first in terms of molecular diagnostic research. In the future, this data, which gives information about whether the embryo will develop in patients who apply to IVF centers without taking a biopsy sample from the embryo, will provide important support to personalized medicine studies. Another importance of the study is that there are not enough data sets on why embryos do not develop in NOR cases. Moreover, this study provides a good molecular data set that sheds light on why NOR embryos do not develop. This study, which we believe will be the starting point for future broad-spectrum related studies, will take a respected place in the scientific literature. For the treatment of diseases and symptoms, the molecules and pathways involved in their pathogenesis must be known. In addition, the expression profiles of the relevant molecules in diseases are also important. Therefore, it is very important to know the molecular pathways that cause pathology before treatment. 

## Figures and Tables

**Table 1:** Gardner’s grading system assigns three distinct quality scores to each blastocyst embryo.

Quality Grade	Blastocyst Stage	Description
1	Early blastocyst	The blastocyst cavity’s volume is less than half of the embryo’s.
2	Blastocyst	The blastocyst cavity is greater than or equal to half of the volume of the embryo
3	Full blastocyst	The blastocyst cavity completely fills the embryo
4	Expanded blastocyst	The volume of the blastocyst cavity is larger than that of the early embryo, and the membrane around it is becoming thinner
5	Hatching blastocyst	Herniation of the outer layer of cells through the surrounding membrane has begun.
6	Hatched blastocyst	Complete separation of the blastocyst from the surrounding membrane occurred.
Blastocyst Structure	Grade	Description
Inner Cell Mass	A	Tightly packed, many cells
Inner Cell Mass	B	Loosely grouped, several cells
Inner Cell Mass	C	Very few cells
Blastocyst Structure	Grade	Description
Trophectoderm	A	Numerous cells creating a closely linked epithelium
Trophectoderm	B	Few cells
Trophectoderm	C	The loose epithelium is made up of very few cells

**Table 2:** Forward and Reverse Primer Sequences used in RT-PCR.

Accession Number	Gene	Forward	Reverse	Tm (Basic)
NM-0024117.1	*Ki-67*	5′-TCCTTTGGTGGGCACCTAAGACCTG-3′	5′-TGATGGTTGAGGTCGTTCCTTGATG-3′	55 °C
AB085628.1	*hTERT*	5′-TGCCAGCCCCAGCGTCAAAG-3′	5′-TGCCAGCCCCAGCGTCAAAG-3′	55.88 °C
OR762216.1	*HIF-1 α*	5′-GGCGCGAACGACAAGAAAAAG-3′	5′-CCTTATCAAGATGCGAACTCACA-3′	54.36 °C
NG-007073.2	*GAPDH*	5′-CGAGGGGGGAGCCAAAAGGG-3′	5′-TGCCAGCCCCAGCGTCAAAG-3′	56 °C

**Table 3:** Correlation relationship between *Ki-67*, *hTERT*, and *HIF-1α* genes in spent embryo culture medium containing developing and non-developing normoresponder embryos. ** indicates *p* < 0.05.

Spent Embryo Culture Medium Containing Developing Normoresponder Embryos				
		***Ki-67***	***hTERT***	***HIF-1α***
*Ki-67*	Pearson correlation	1	−0.364	0.313
	Sig. (2-tailed)		0.115	0.179
	N	20	20	20
*hTERT*	Pearson correlation	−0.364	1	−0.021
	Sig. (2-tailed)	0.115		0.931
	N	20	20	20
*HIF-1α*	Pearson correlation	0.313	−0.021	1
	Sig. (2-tailed)	0.179	0.931	
	N	20	20	20
Spent Embryo Culture Medium Containing Non-Developing Normoresponder Embryos				
		***Ki-67***	***hTERT***	***HIF-1α***
*Ki-67*	Pearson correlation	1	−0.181	0.762 **
	Sig. (2-tailed)		0.444	0.000
	N	20	20	20
*hTERT*	Pearson correlation	−0.181	1	0.123
	Sig. (2-tailed)	0.444		0.605
	N	20	20	20
*HIF-1α*	Pearson correlation	0.762 **	0.123	1
	Sig. (2-tailed)	0.000	0.605	
	N	20	20	20

## Data Availability

Data sets generated during and analyzed during the current study are available from the corresponding author on reasonable request.

## Supplementary Materials

The following supporting information can be downloaded at: https://www.mdpi.com/article/10.3390/cells13242093/s1, Supplementary Materials S1: The statistical analysis of the fold change of Ki-67 (*p* > 0.05), hTERT (*p* < 0.05), and HIF-1α (*p* < 0.001) gene expressions which were determined in developing and non-developing embryo spent culture medium from normoresponder (NOR) patients.
