# Supplementary material for: Molecular Markers in Embryo Non-Development: Analysis of Gene Expressions (Ki-67, hTERT, HIF-1α) in Spent Embryo Culture Medium
Source: Cells. 2024 Dec 18;13(24):2093. doi: 10.3390/cells13242093 (PMC11674905; doi:10.3390/cells13242093)
Supplement: Supplementary file 1 [file cells-13-02093-s001.zip › cells-3277211-supplementary.pdf]

Tests of Normality

	GRUP	Kolmogorov-Smirnov[a] Statistic	df	Sig.	Shapiro-Wilk Statistic	df	Sig.
Ki_67	GELİŞEN	,190	20	,057	,935	20	,195
	GELİŞMEYEN	,192	20	,051	,874	20	,014
Htert	GELİŞEN	,140	20	,200*	,961	20	,558
	GELİŞMEYEN	,176	20	,106	,934	20	,184
HIF_1α	GELİŞEN	,180	20	,088	,945	20	,302
	GELİŞMEYEN	,178	20	,097	,878	20	,016

*. This is a lower bound of the true significance.

a. Lilliefors Significance Correction

T-Test

Group Statistics

	GRUP	N	Mean	Std. Deviation	Std. Error Mean
Ki_67	GELİŞEN	20	5,9985	1,75863	,39324
	GELİŞMEYEN	20	6,2351	2,87910	,64379
Htert	GELİŞEN	20	-1,0223	2,27460	,50862
	GELİŞMEYEN	20	-2,7592	1,90447	,42585
HIF_1α	GELİŞEN	20	9,0108	1,33564	,29866
	GELİŞMEYEN	20	11,2988	1,68867	,37760

Independent Samples Test

		Levene's Test for Equality of Variances F	Sig.	t-test for Equality of Means t	df	Sig. (2-tailed)
Ki_67	Equal variances assumed	1,919	,174	-,314	38	,755
	Equal variances not assumed			-,314	31,446	,756
Htert	Equal variances assumed	,314	,579	2,618	38	,013
	Equal variances not assumed			2,618	36,861	,013
HIF_1α	Equal variances assumed	,285	,597	-4,752	38	,000
	Equal variances not assumed			-4,752	36,086	,000

Correlations

GRUP = GELİŞEN

Correlations[a]

		Ki_67	Htert	HIF_1α
Ki_67	Pearson Correlation	1	-,364	,313
	Sig. (2-tailed)		,115	,179
	N	20	20	20
Htert	Pearson Correlation	-,364	1	-,021
	Sig. (2-tailed)	,115		,931
	N	20	20	20
HIF_1α	Pearson Correlation	,313	-,021	1
	Sig. (2-tailed)	,179	,931	
	N	20	20	20

a. GRUP = GELİŞEN

GRUP = GELİŞMEYEN

Correlations[a]

		Ki_67	Htert	HIF_1α
Ki_67	Pearson Correlation	1	-,181	,762**
	Sig. (2-tailed)		,444	,000
	N	20	20	20
Htert	Pearson Correlation	-,181	1	,123
	Sig. (2-tailed)	,444		,605
	N	20	20	20
HIF_1α	Pearson Correlation	,762**	,123	1
	Sig. (2-tailed)	,000	,605	
	N	20	20	20

**. Correlation is significant at the 0.01 level (2-tailed).

a. GRUP = GELİŞMEYEN
